# Supplementary material for: The orchestrating role of platform enterprises in digital inclusion: A network game perspective from industrial clusters
Source: PLoS One. 2026 Apr 1;21(4):e0336724. doi: 10.1371/journal.pone.0336724 (PMC13042771; doi:10.1371/journal.pone.0336724)
Supplement: S2 Table — (PDF) [file pone.0336724.s002.pdf]

S2 Table. List of Parameters.

Parameter Symbol	Meaning
n	Total number of enterprises (nodes) in the industrial cluster
I_0	Initial industrial cluster network
P_0	Set of enterprises (nodes) in the initial network
D_0	Set of directed edges in the initial network
p_i	The i-th enterprise (node) in the network
d_{ij}	Directed edge, from node p_i to node p_j
P_i	Set of neighbour nodes that receive business from enterprise p_i
d_i	Total number of neighbour nodes that receive business from p_i
I	The network after integrating the platform enterprise
p_{n+1}	The platform enterprise node
P	Set of nodes after integrating the platform enterprise
D	Set of directed edges after integrating the platform enterprise
$PR(i)$	The eigenvector centrality of firm p_i, which represents the importance of node p_i within the network.
γ	The damping factor of the PageRank algorithm.
$E(i)$	A decay factor, introduced to prevent the accumulation of PageRank (PR values) that would otherwise occur if the transmitted PR remained trapped within clusters of nodes lacking outbound links.
σ	Average out-degree of nodes in the initial network
q	Edge rewiring probability in the small-world network
λ_i	Strategy of enterprise p_i
c	Single-round investment cost for an enterprise
r	Investment return coefficient
π_i	Single-round game payoff for enterprise p_i
$R_{i\leftarrow j}$	Probability that enterprise p_i imitates enterprise p_j
k	Noise parameter in the Fermi function
T	Total number of Monte Carlo simulation runs
λ_{n+1}	Strategy of the platform enterprise
$P(x_t)$	Proportion of co-constructing enterprises (excluding the platform) in the t-th round
P_y	Monte Carlo average of the proportion of co-constructing enterprises
x	Number of game iterations
t	The t-th Monte Carlo simulation run
