# Supplementary material for: The orchestrating role of platform enterprises in digital inclusion: A network game perspective from industrial clusters
Source: PLoS One. 2026 Apr 1;21(4):e0336724. doi: 10.1371/journal.pone.0336724 (PMC13042771; doi:10.1371/journal.pone.0336724)
Supplement: Attachment — Submitted filename: Response to Reviewers.docx [file pone.0336724.s004.docx]

**Response to the Editors and Reviewers**

Manuscript ID: PONE-D-25-58355

Manuscript Title: The Orchestrating Role of Platform Enterprises in Digital Inclusion: A Network Game Perspective from Industrial Clusters

Dear Editors and Reviewers,

Thank you for handling our manuscript and for providing highly constructive feedback. We have carefully reviewed and considered all suggestions and have undertaken a systematic revision of the manuscript accordingly. Below, we provide a point-by-point response detailing the changes made.

**1. Responses to Journal Formatting and Policy Requirements**

1.1 Formatting and File Naming

We have carefully formatted the manuscript according to the PLOS ONE template and have prepared the three required files:

"Response to Reviewers"

"Revised Manuscript with Track Changes"

"Manuscript" (Clean version)

1.2 Code Sharing

The simulation code for this study was developed in MATLAB (version 2022b or later). To ensure reproducibility, the code has been uploaded to the Open Science Framework (OSF) and is available at the following link:

https://osf.io/axtf9/overview?view_only=f39e90a6a71e40cd811aa5469a4aa9f6

Detailed run instructions are provided. The code has also been uploaded as supplementary material to the PLOS ONE submission system.

1.3 Data Availability Statement

The Data Availability Statement has been updated to confirm that all simulation data and code are accessible via the OSF repository. The data underlying the figures have been provided as Supporting Information files. Furthermore, the simulation data files have been uploaded to the PLOS ONE submission system. The .fig files can be opened in MATLAB to view the detailed data.

1.4 Financial Disclosure Statement

The Guangxi Science and Technology Think Tank Key Project (Grant No. K‑88) funded computational resource leasing.The Guangxi Basic Ability Improvement Project for Young and Middle‑aged Teachers in Universities (Grant No. 2024KY1937) covered publication-related expenses.The Guangxi Vocational Education Teaching Reform Research Project (Grant No. GXGZJG2024A004) supported costs associated with literature retrieval, access, and research-related travel.The funders had no role in study design, data collection and analysis, decision to publish, or preparation of the manuscript.

1.5 Acknowledgments Section

All funding-related acknowledgments have been removed from this section, retaining only thanks to the institution that provided computational support.

1.6 No specific citations are required.

The reviewers' comments do not include recommendations to cite specific previously published works.

**2. Response to Reviewer #1**

We thank the reviewer for their valuable comments aimed at enhancing scientific transparency, reproducibility, and the clarity and structure of the methodological description.

In direct response:

All model code and simulation data have been made publicly available on OSF (link provided in Section 1.2).The same code and data have also been uploaded as supplementary files to the PLOS ONE submission system for convenience.While the code is complex, we have added comprehensive comments throughout to aid understanding of the model's framework and logic.

We believe these actions significantly improve the transparency and replicability of our study. We are grateful for the reviewer's insights and are ready to provide further clarification if needed.

**3. Response to Reviewer #2**

We sincerely thank the reviewer for their insightful comments regarding conceptual clarity and interpretive coherence. To address this core concern, we have added a synthesising overview paragraph at the end of the Introduction section. This addition explicitly integrates the key components of our theoretical framework and analytical approach, thereby strengthening the logical flow and narrative consistency throughout the manuscript.

We appreciate the reviewer's guidance in enhancing the manuscript's readability and theoretical rigour. We are happy to provide further clarification on any specific points if required.

Sincerely,

Corresponding Author: Dingteng Wang
