# Supplementary material for: The orchestrating role of platform enterprises in digital inclusion: A network game perspective from industrial clusters
Source: PLoS One. 2026 Apr 1;21(4):e0336724. doi: 10.1371/journal.pone.0336724 (PMC13042771; doi:10.1371/journal.pone.0336724)
Supplement: Attachment — Submitted filename: Response to ReviewersV2.docx [file pone.0336724.s005.docx]

****Response to Reviewers****

****Manuscript ID:**** PONE-D-25-58355R1
****Title:**** The orchestrating role of platform enterprises in digital inclusion: A network game perspective from industrial clusters

Dear Editor and Reviewers,

We would like to thank the editor and the reviewers for their thoughtful and constructive comments on our revised manuscript. We are pleased that Reviewer #2 acknowledges the manuscript as "technically sound" and suitable for publication following minor revisions, and we appreciate Reviewer #1's continued engagement in improving the methodological clarity and transparency of our work.

We have carefully addressed all remaining concerns raised by both reviewers. Below, we provide a point-by-point response outlining the revisions made.

**Response to Reviewer #1**

We thank Reviewer #1 for emphasising the importance of methodological transparency and conceptual clarity. In response, we have made the following revisions:

**Clarification of the simulation-based nature of the study**: In both the **Abstract** and **Introduction**, we have explicitly stated that this is a **theoretical simulation study** and that the data used are **synthetically generated through Monte Carlo simulations**. We have also included a description of the **"Monte Carlo steps"** to improve procedural transparency.

**Enhancement of result robustness**: To demonstrate the stability of our findings, we have added **confidence intervals** to all figures. Additionally, we have uploaded the **raw data in .xlsx format** to the **OSF public repository** and the **PLOS ONE submission system** to facilitate full reproducibility.

**Improvement of methodological transparency**: We have included a new **Fig 1. Flowchart of the game procedure** to visually illustrate the simulation process. We have also corrected several parameter inconsistencies and provided a **detailed parameter table** in the **Appendix**, specifying the rationale and literature basis for each parameter choice. Confirmed that all data and code are accessible via the provided OSF link.

**https://osf.io/axtf9/overview?view_only=f39e90a6a71e40cd811aa5469a4aa9f6**

We hope these revisions adequately address your concerns and enhance the clarity and rigour of the manuscript.

**Response to Reviewer #2**

We are grateful for your positive assessment and your recognition that the manuscript is now technically sound. We also appreciate your helpful suggestions for final polishing. In response, we have made the following editorial improvements:

**Conceptual consistency**: We have systematically reviewed the manuscript to ensure consistent use of key terminology throughout, including terms such as "platform enterprise," "investment strategy," "free-rider," and "connection ratio (CR)."

**Language and readability**: We have carefully proofread the manuscript and refined the language in several sections, particularly in the discussion, to improve clarity and fluency.

**Consistency in presentation**: We have revised all figures for visual consistency, standardised notation across the manuscript, and provided a comprehensive **parameter table** in the **Appendix** to complement the methodological description.

We believe these revisions have further strengthened the manuscript and we thank you once again for your constructive feedback.

We hope that the revised manuscript now meets the standards for publication in PLOS ONE and we look forward to your final decision.

Thank you for your time and consideration.
